# Validation and Feasibility of Echocardiographic Assessment of Systemic Right Ventricular Function: Serial Correlation With MRI

**DOI:** 10.3389/fcvm.2021.644193

**Published:** 2021-03-16

**Authors:** Tjitske E. Zandstra, Monique R. M. Jongbloed, Ralph L. Widya, Arend D. J. ten Harkel, Eduard R. Holman, Bart J. A. Mertens, Hubert W. Vliegen, Anastasia D. Egorova, Martin J. Schalij, Philippine Kiès

**Affiliations:** ^1^Department of Cardiology, Leiden University Medical Center, Leiden, Netherlands; ^2^Department of Anatomy and Embryology, Leiden University Medical Center, Leiden, Netherlands; ^3^Department of Radiology, Leiden University Medical Center, Leiden, Netherlands; ^4^Department of Paediatrics, Leiden University Medical Center, Leiden, Netherlands; ^5^Department of Biomedical Data Sciences, Leiden University Medical Center, Leiden, Netherlands

**Keywords:** echocardiography, magnetic resonance imaging, transposition of great vessels, observer variation, congenital heart disease

## Abstract

**Background:** Inherent to its geometry, echocardiographic imaging of the systemic right ventricle (RV) is challenging. Therefore, echocardiographic assessment of systemic RV function may not always be feasible and/or reproducible in daily practice. Here, we aim to validate the usefulness of a comprehensive range of 32 echocardiographic measurements of systemic RV function in a longitudinal cohort by serial assessment of their correlations with cardiac magnetic resonance (CMR)-derived systemic RV ejection fraction (RVEF).

**Methods:** A single-center, retrospective cohort study was performed. Adult patients with a systemic RV who underwent a combination of both CMR and echocardiography at two different points in time were included. Off-line analysis of echocardiographic images was blinded to off-line CMR analysis and vice versa. In half of the echocardiograms, measurements were repeated by a second observer blinded to the results of the first. Correlations between echocardiographic and CMR measures were assessed with Pearson's correlation coefficient and interobserver agreement was quantified with intraclass correlation coefficients (ICC).

**Results:** Fourteen patients were included, of which 4 had congenitally corrected transposition of the great arteries (ccTGA) and 10 patients had TGA late after an atrial switch operation. Eight patients (57%) were female. There was a mean of 8 years between the first and second imaging assessment. Only global systemic RV function, fractional area change (FAC), and global longitudinal strain (GLS) were consistently, i.e., at both time points, correlated with CMR-RVEF (global RV function: *r* = −0.77/*r* = −0.63; FAC: *r* = 0.79/*r* = 0.67; GLS: *r* = −0.73/*r* = −0.70, all *p*-values < 0.05). The ICC of GLS (0.82 at *t* = 1, *p* = 0.006, 0.77 at *t* = 2, *p* = 0.024) was higher than the ICC of FAC (0.35 at *t* = 1, *p* = 0.196, 0.70 at *t* = 2, *p* = 0.051) at both time points.

**Conclusion:** GLS appears to be the most robust echocardiographic measurement of systemic RV function with good correlation with CMR-RVEF and reproducibility.

## Introduction

**Figure 1:**
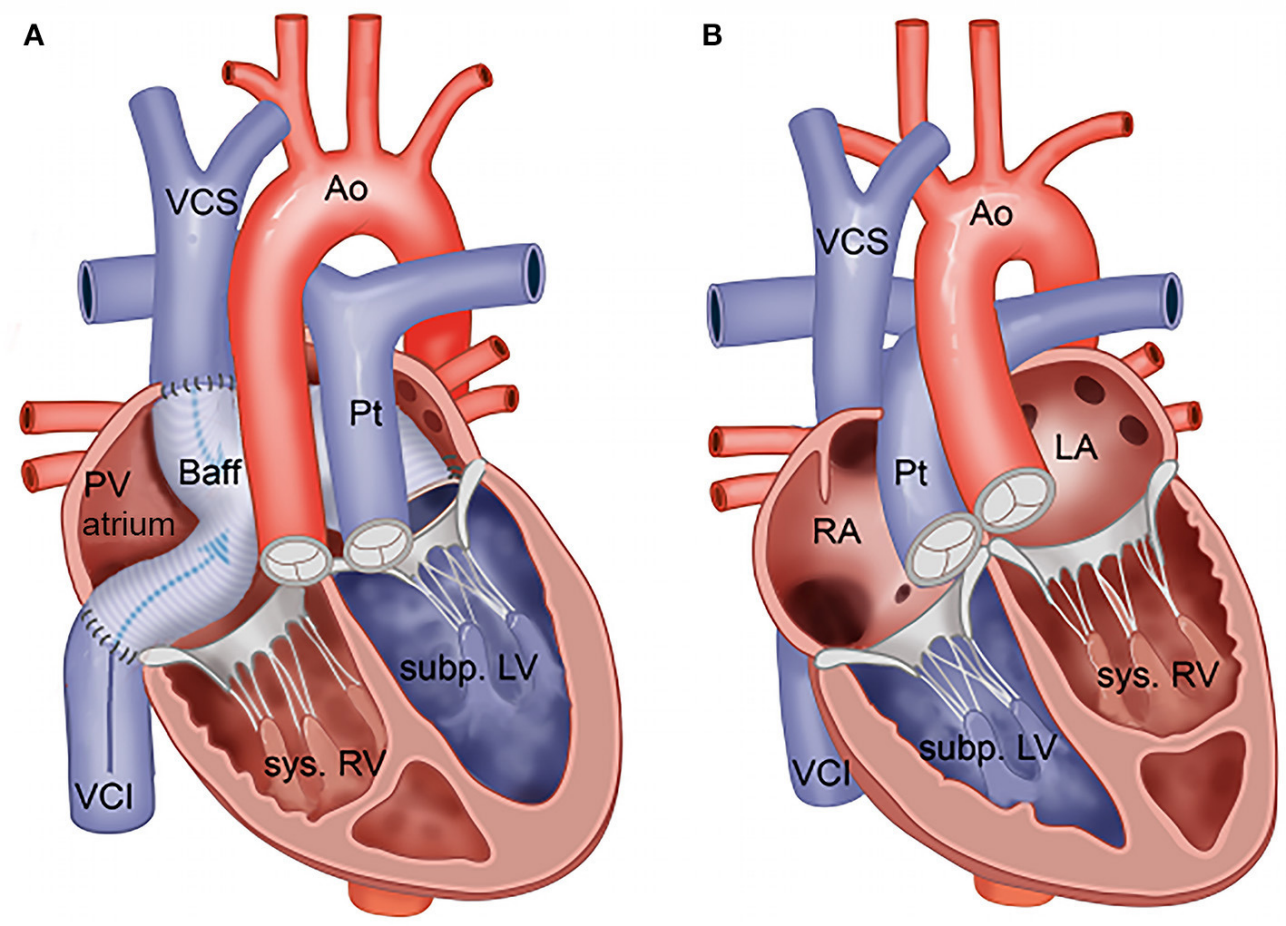
**(A)** Transposition of the great arteries after Mustard or Senning repair. **(B)** Congenitally corrected transposition of the great arteries. Ao, aorta; Baff, systemic venous baffle; LA, (morphologically) left atrium; Pt, pulmonary trunk; PV atrium, pulmonary venous atrium; RA, (morphologically) right atrium; Subp. LV, subpulmonary LV; Sys. RV, systemic RV; VCI, inferior vena cava; VCS, superior vena cava.

In congenital heart disease with a systemic right ventricle (RV), the RV supports systemic circulation. This includes transposition of the great arteries (TGA) after atrial switch operation and congenitally corrected TGA (ccTGA) (Figure 1). The systemic RV is morphologically and functionally different from the left ventricle (LV) and is more suitable to process volume than pressure. Systemic RV failure is a well-known, long-term complication as are tricuspid valve regurgitation, conduction abnormalities, and arrhythmias (1, 2). Progression from subclinical to clinical systemic RV dysfunction can be sudden and unexpected, hampering identification of the ideal window of intervention. Early and reliable detection of reduced systemic RV function is essential but challenging for several reasons. The shape of the systemic RV limits the possibilities for calculations based on spatial assumptions, such as ejection fraction (3), and pronounced trabeculations impair the measurement of the volume of the systemic RV. Cardiac magnetic resonance (CMR) imaging is regarded as the gold standard for volumetric and functional assessment of systemic RV (4, 5) although it is often not feasible because patients may have pacemakers or implantable cardioverter defibrillators with epicardial or abandoned leads. Thus, transthoracic echocardiography (TTE) is still the main tool for the assessment of systemic RV function in clinical practice (3). Previous studies evaluated echocardiographic indices of systemic RV function, such as global longitudinal strain (GLS), fractional area change (FAC), isovolumic acceleration (IVA), the myocardial performance index (MPI), and tricuspid annular plane systolic excursion (TAPSE), which may all have value in the evaluation of systemic RV function (6–15).

There is a gap between the research setting and daily clinical practice: systemic RV geometry in combination with previous thoracotomies makes specific echocardiographic views difficult to obtain. Reported correlations between echocardiographic variables and CMR-derived RVEF differ (3), and interobserver variability may influence the reliability of individual measurements (11, 16, 17). No studies have evaluated the consistency of the correlation between both imaging techniques over time.

This study aims to distill echocardiographic variables that are feasible to obtain in daily clinical practice, are consistently correlated with CMR-derived RVEF, and can be measured reliably.

## Methods

We aimed to validate the usefulness of a comprehensive range of echocardiographic variables of systemic RV function in a single-center cohort. Echocardiographic variables were compared with CMR-RVEF at two different points in time. All reported echocardiographic variables of systemic RV function were included. When applicable, different methods of evaluating a single variable were compared. Interobserver agreement and feasibility of the variables were assessed.

### Study Design and Patient Selection

A retrospective cohort study was conducted. Adult patients with a systemic RV in a biventricular circulation (TGA after Mustard or Senning procedure or ccTGA) from the outpatient congenital cardiology clinic of the Leiden University Medical Center were screened for availability of a combination of both CMR and TTE images at two different points in time at least 5 years apart. The CMR and TTE images at the same points in time should be no more than 1 year apart. All consecutive patients meeting these criteria were included. Complex (cc)TGA was defined as (cc)TGA with important additional malformations present at birth, including ventricular septal defects, left ventricular outflow tract (LVOT) obstruction, and aortic arch malformations. The Leiden University Medical Center's medical research ethics committee waived the need for informed consent. The protocol is in accordance with the 2013 Declaration of Helsinki.

### Selection of Echocardiographic Variables

To identify a comprehensive range of echocardiographic variables of systemic RV function, a PubMed search was conducted with the following key terms: “systemic right ventricle,” “(congenitally corrected) transposition of the great arteries,” “echocardiography,” “atrial switch,” “Mustard,” “Senning.” From the articles found, studies in which echocardiography was conducted in cohorts of patients with a systemic RV (TGA after atrial switch procedure, ccTGA, or both) were screened for echocardiographic variables reflecting systemic RV function. Both studies in which echocardiographic variables were compared with CMR-RVEF and studies assessing associations between echocardiographic variables and clinical variables (such as exercise capacity) or clinical events/outcomes were screened. From these studies, all variables that could be reasonably measured in our cohort were added to the protocol.

### Echocardiography

Clinically indicated echocardiograms performed with commercially available ultrasound systems were analyzed off-line in EchoPac, GE Medical Systems. Length, weight, and heart rate at the time of imaging were noted. Measurements were performed off-line by one researcher (TZ) who received training from three experienced congenital imaging cardiologists: PK (European Association of Cardiovascular Imaging [EACVI] certified for >10 years), DH (EACVI member), and EH (head of the imaging lab, supervisor of EACVI candidates). To assess interobserver agreement, measurements were repeated by PK in half of the echocardiograms, blinded to the measurements of TZ. Both observers were blinded to the MRI measurements. Table 1 describes the variables that were measured or calculated. If several methods to calculate the same variable were available, all were applied to allow comparison (e.g., MPI). In total, 32 variables of systemic RV function were assessed. For completeness, atrial dimensions and subpulmonary LV function variables were additionally assessed.

**Table 1:** Echocardiographic variables studied.

**Variable**	**Specification**	**Unit**	**Reference values**
**DIMENSIONS**			
LA length^a^	Left atrial end-systolic length	mm	
LA volume index (LAVI)^a^	Left atrial end-systolic volume (Simpson's biplane method) indexed by body size	mL/m^2^	
RA length^a^	Right atrial end-systolic length	mm	
RV apex-base length	RV end-diastolic length	mm	84 mm (11)
Mid-RV diameter	RV end-diastolic diameter at half of the apex-base length	mm	
RV base diameter	Maximal RV end-diastolic diameter in basal 1/3 of the ventricle	mm	49 mm (11); 47 mm (12); 45.6 mm (18)
RV free wall thickness	End-diastolic thickness in PLAX	mm	10 mm (18)
**SYSTEMIC RV FUNCTION**			
RV global function	Visual assessment of systemic right ventricular function	Normal, mildly abnormal, moderately abnormal, severely abnormal	62% moderately or severely abnormal (11)
RV FAC	Fractional area change: (RV end-diastolic area-RV end-systolic area)/RV end-diastolic area*100%	%	31.7% (19); 24% (11); 32% (12); 23% (20); 22.9% (18); 38.7% (9); 44%• (14); 38% (15)
RV GLS	Maximum global longitudinal strain in AP4CH	%	−13.3% (21); −14.2% (11); −12% (20); −13.5% (16); −12.5% (18); −18.7%• (14); −14.6% (15); −15.5% (12)
RV SR S	Maximum global systolic strain rate	%/s	−0.61%/s (15); −0.59 (18)
RV SR E	Maximum global strain rate during early diastole	%/s	0.68 (18)
RV SR A	Maximum global strain rate during late diastole	%/s	0.32 (18)
RV septal strain	Maximum mid-septal longitudinal strain in AP4CH	%	−12.2% (11); −12.1 (15)
RV septal SR S	Maximum mid-septal systolic strain rate	%/s	
RV septal SR E	Maximum mid-septal strain rate during early diastole	%/s	
RV septal SR A	Maximum mid-septal strain rate during late diastole	%/s	
RV free wall strain	Maximum mid-lateral longitudinal strain in AP4CH	%	−14.7% (11); −15.0 (15)
RV free wall SR S	Maximum mid-lateral systolic strain rate	%/s	−1.06%/s (11)
RV free wall SR E	Maximum mid-lateral strain rate during early diastole	%/s	
RV free wall SR A	Maximum mid-lateral strain rate during late diastole	%/s	
IVA PW-TDI AP4CH	Isovolumic acceleration: V_max_/time to V_max_ during isovolumetric acceleration	m/s^2^	
IVA TDI AP4CH	Isovolumic acceleration: V_max_/time to V_max_ during isovolumetric acceleration	m/s^2^	0.9 m/s^2^ (15); 1.33 (18)
MPI PW-TDI AP4CH	Myocardial performance index (Isovolumetric contraction time + isovolumetric relaxation time)/ejection time	–	
MPI TDI AP4CH	Myocardial performance index (Isovolumetric contraction time + isovolumetric relaxation time)/ejection time	–	0.41 (15); 0.53 (19)
MPI TV inflow/RV outflow	Myocardial performance index (Isovolumetric contraction time + isovolumetric relaxation time)/ejection time	–	0.57 (14); 0.47 (9); 0.63 (18)
MPI CW-TR/RV outflow	Myocardial performance index (Isovolumetric contraction time + isovolumetric relaxation time)/ejection time	–	
Tricuspid valve lateral velocity	Velocity measured with PW-TDI	cm/s	7.2 (8); 9 (12); 8 (15); 9.1 (14); 9.7 (9); 5.1 (18); 8 (16); 8.4 (11); 5.2 (19)
Tricuspid valve septal velocity	Velocity measured with PW-TDI		
TAPSE	Tricuspid annular plane systolic excursion	mm	12 mm (11); 12 mm (20); 13 mm (16); 9.8 mm (18); 14.3 mm (9); 16.4 mm• (14) 12.5 mm (15); 14 mm (12); 13 mm (8)
MAPSE septal	Mitral annular plane systolic excursion (septal aspect)		
Tricuspid valve dP/dt	Continuous wave Doppler tricuspid regurgitation: time between 1 and 3 m/s (=time necessary for RV pressure to increase 32 mmHg)	mmHg/s	1,625 (12); 868 (15); 1,167 (9); 1,024 (16); 833 (11);
Tricuspid valve regurgitation	Based on integration of a.o. jet density/contour, vena contracta width, and proximal isovelocity surface area-radius [see (22)]	Mild, moderate, severe	> mild: 40% (11); 63% (20); 33% (16)
**DYSSYNCHRONY**			
Intraventricular delay (strain)	Time to peak strain RV free wall–time to peak strain RV septal wall	ms	48 ms (19)
Interventricular delay (strain)	Time to peak strain RV free wall—time to peak strain LV free wall	ms	63 ms (19)
Interventricular delay (output)	Time between Q wave and start output RVOT in CW Doppler—Time between Q wave and start output LVOT in CW Doppler	ms	50 ms (23)
**DIASTOLIC FUNCTION SYSTEMIC RV**			
Tricuspid valve E/A ratio		–	1.7 (8)
Tricuspid valve E/e' ratio		–	7.6 (8)
**LEFT VENTRICULAR DIMENSIONS AND FUNCTION**			
Left ventricular end diastolic diameter	Measured in PLAX and AP4CH	mm	34 (14); 32 (19)
LV GLS	Maximum global longitudinal strain in AP4CH		−18.6% (21)
MAPSE	Mitral annular plane systolic excursion	mm	21.8 mm• (14); 19 mm (8)

a *only for patients with congenitally corrected transposition of the great arteries; AP4CH, apical four-chamber view; CW doppler, continuous wave doppler; FAC, fractional area change; GLS, global longitudinal strain; IVA, isovolumic acceleration; LA, left atrial; LV, left ventricle; LVOT, left ventricular outflow tract; MAPSE, mitral annular plane systolic excursion; MPI, myocardial performance index; PLAX, parasternal long-axis view; PW-TDI, pulsed-wave tissue doppler imaging; RA, right atrial; RV, right ventricle; RVOT, right ventricular outflow tract SR A, maximal late diastolic strain rate; SR E, maximal early diastolic strain rate; SR S, maximal systolic strain rate; TAPSE, tricuspid annular plane systolic excursion*.

*(21): N = 129, Mustard/Senning/ccTGA, 31% NYHA>1. Mean RVEF 52%. (11): N = 42, Mustard/ccTGA, median NT-pro-BNP 27.4 pmol/L (232 ng/L) (20): N = 105, Mustard/Senning/ccTGA, 29% NYHA>1. Mean RVEF 42%, mean percent predicted peak VO_2_ 69%. (16): N = 35, Mustard/Senning. Twelve percent NYHA>1. Mean RVEF 44%. (18): N = 64, Mustard/Senning. Optimal cutoff for prediction of events (incident heart failure or ventricular tachycardia): GLS−10% (9): N = 37, Mustard/Senning. Twenty-two percent NYHA>1. Optimal cutoff for CMR-RVEF 50%: FAC 33%. (14): N = 33, ccTGA, 36% NYHA>1. Optimal cutoff for CMR-RVEF 45%: GLS−16.3%. •: values given for the part of the group with CMR-derived RVEF≥45%; (15): N = 47, Mustard/Senning, 13% NYHA>1. Median percent predicted peak VO_2_ value 64.5%. Mean RVEF 47%. (12): N = 48, Mustard/Senning, mean RVEF: 48%. Optimal cut-off for RVEF 45%: FAC 29.5% and GLS−14.2%. (8): N = 46, Mustard/Senning/ccTGA, 24% NYHA>1. Median NT-pro-BNP 475 ng/L (23): N = 8, Mustard/Senning/ccTGA/DORV, undergoing CRT. Values given measured after CRT. (19): N = 28, Mustard/Senning, mean FAC = 31.7*.

Global systemic RV function was visually assessed from the apical four-chamber view and parasternal long and short axis views. Systemic RV free wall thickness was measured in end-diastole in the parasternal long axis view. Systemic RV dimensions and areas and subpulmonary LV dimensions were measured in the apical four-chamber view in all patients. Trabeculations were included in the cavum. FAC was calculated as the percentage of change between the end-diastolic and end-systolic areas. Speckle tracking GLS and strain rate (SR) were determined in the apical four-chamber view by the software package in EchoPac after manual determination of the endocardial border (24). The placement of the automatically allocated markers was adjusted manually after visual inspection during movement to include the entire myocardium, including the free wall, apex, and septum. GLS was determined for both the systemic RV and subpulmonary LV. For the systemic RV, longitudinal strain and SR were also calculated separately for the mid-free wall and mid-septal regions. TAPSE and mitral annular plane systolic excursion (MAPSE) were determined in M-mode by placing the cursor in the lateral aspect of, respectively, the tricuspid and mitral valves. The effective regurgitant volume of the tricuspid valve was determined with the proximal isovelocity surface area method in the color doppler and continuous wave doppler images of the tricuspid valve (25)_._ Early and late diastolic velocities were measured from the pulsed wave doppler image of the tricuspid valve inflow. The rate of pressure build-up in the systemic RV (dP/dt) was calculated from the continuous wave image of tricuspid regurgitation in the interval for the regurgitation velocity to increase from 1 to 3 m/s. Tricuspid valve regurgitation itself was assessed semiquantitatively based on integration of several variables in accordance with the ESC recommendations as described elsewhere (22). The MPI was calculated as (isovolumic contraction time + isovolumic relaxation time)/ejection time. In patients with reduced ventricular function, the isovolumic contraction and relaxation times are longer and the ejection time shorter. Thus, a higher MPI reflects reduced ventricular function (26). MPI was calculated in four ways (Supplementary Figure 1): first, from the pulsed wave tissue doppler image of the tricuspid valve in the apical four-chamber view and, second, from manual analysis of the tissue doppler image. Third, it was calculated from the continuous or pulsed wave doppler image of the tricuspid valve inflow and the continuous or pulsed wave doppler image of the RV outflow tract. Last, it was calculated from the continuous wave signal of tricuspid valve regurgitation and the continuous or pulsed wave doppler image of the RV outflow tract. The first two methods only require one image, which limits variation because of heart rate but only uses the motion of the lateral aspect of the tricuspid valve and, therefore, may have the disadvantage of reflecting regional rather than global ventricular performance. The third and fourth methods may reflect ventricular performance more globally but have the disadvantage that measurements have to be performed in separate images, allowing differences in heart rate to introduce variation (27).

The isovolumic acceleration was calculated as the slope of velocity increase of the lateral aspect of the tricuspid valve during isovolumic contraction (28). It was calculated from the pulsed wave tissue doppler image of the lateral tricuspid valve in the apical four-chamber view and from the tissue doppler image in which the cursor was manually placed at the lateral aspect of the tricuspid valve.

Systolic and early and late diastolic velocities of the lateral aspect of the tricuspid valve and systolic velocity of the septal aspect of the tricuspid valve were measured in the pulsed wave tissue doppler and the tissue doppler apical four-chamber images.

Intraventricular dyssynchrony was calculated as the difference between the time to peak strain in speckle tracking analysis between the systemic RV mid-lateral and mid-septal wall (16, 19, 29). Interventricular dyssynchrony was calculated in two ways: as the difference between time to peak strain between the systemic RV mid-free wall and the subpulmonary LV mid-free wall and as the difference between onset of Q-start ejection between the RVOT and LVOT (30).

### Cardiac Magnetic Resonance Imaging

MRI studies were performed with a 1.5-T whole-body MRI scanner (Philips Medical Systems, Best, the Netherlands). The routine clinical protocol included electrocardiographically gated breath-hold, steady-state, free precession imaging in transverse orientation. Length, weight, and heart rate at the time of imaging were noted. To assess cardiac dimensions and systolic function, end-diastolic and end-systolic endocardial contours were manually drawn with software (MASS; Medis Medical Imaging Systems, Leiden, the Netherlands) by an experienced cardiothoracic radiologist (RW) who was blinded to the echocardiographic measurements. Trabeculations were included in the cavity. Systemic RV dimensions and free wall thickness were measured in end-diastole in the four-chamber view. Stroke volume was calculated by subtracting the end-systolic from the end-diastolic volume. Systemic right ventricular ejection fraction (RVEF) was calculated as the percentage of volume change between the end-diastolic and end-systolic volumes. Cardiac output was calculated by multiplying the stroke volume by heart rate.

### Statistical Analysis

For all analyses, IBM SPSS statistics 25 was used. Data are presented as mean ± standard deviation (SD) (or median and interquartile range [IQR] as appropriate) or frequencies and percentages. Changes between variables over time were tested with Student's *T*-test. Correlations between echocardiographic and CMR imaging variables were tested with Pearson's or Spearman's correlation analysis as appropriate. Interobserver agreement was visually assessed by calculation of the mean difference between observed values and constructing the limits of agreement (±1.96 SD of the difference, thus including 95% of measurements) according to Bland and Altman (31). Interobserver agreement was statistically assessed with calculation of intraclass correlation coefficients (ICC). Agreement between systemic RV dimensions as measured on the echocardiograms and the CMR images was also assessed with calculation of the ICC. All correlations and ICCs were calculated separately for the two points in time. *P*-values of < 0.05 were considered statistically significant.

## Results

### Patient Characteristics

Fourteen patients were included. The majority of patients underwent a Mustard or Senning procedure for TGA. Heart rate and body surface area were not significantly different between the first and the second time point. The QRS duration increased between the first and second time point; however, few patients had a QRS duration > 130 ms (1 at *T* = 1 and 2 at *T* = 2). The overall New York Heart Association (NYHA) class worsened between the first and second time points (Table 2).

**Table 2:** Patient characteristics and imaging values at *T* = 1 and *T* = 2.

	***T* = 1**	***T* = 2**	
**Clinical characteristics**	**Mean ± SD, median [IQR], or *N* (%)**	**Mean ± SD, median [IQR], or *N* (%)**	***p*-value**
Female	8 (57%)		
ccTGA	4 (29%)		
Mustard/Senning	10 (71%)		
Complex (cc)TGA	9 (64%)		
VSD^a^	0 (0%)	0 (0%)	
LVOT stenosis^a^	4 (29%)	4 (29%)	
Prior TVR/TVP	0 (0%)	0 (0%)	
Age	35 ± 7	43 ± 7	
QRS duration (ms)	106 [92–116]	112 [108–119]	0.038^*^
Heart rate (bpm)	70 ± 17	69 ± 15	0.342
Rhythm			1,000
Sinus rhythm	13 (93%)	12 (86%)	
Atrial rhythm	1 (7%)	2 (14%)	
BSA (m^2^)	1.9 [1.8–2.0]	1.9 [1.8–2.0]	0.638
NYHA class			0.025^*^
I	7 (50)	2 (14)	
II	6 (43)	11 (79)	
III	1 (7)	1 (7)	
**ECHOCARDIOGRAPHIC VARIABLES**			
RV apex base diameter (mm)	77 ± 9	80 ± 9	0.011^*^
RV mid diameter (mm)	43 ± 9	45 ± 7	0.230
RV basal diameter (mm)	50 ± 6	52 ± 5	0.749
RV free wall thickness (mm)	8 ± 2	9 ± 4	0.432
RV FAC (%)	27 ± 7	24 ± 5	0.071
RV global function moderately or severely reduced (*N*, %)	3 (21%)	4 (29%)	0.317
RV GLS (%)	−14.5 ± 3.0	−15.0 ± 2.7	0.508
TAPSE (mm)	12 ± 3	14 ± 2	0.304
Tricuspid regurgitation > mild (*N*, %)	5 (36%)	4 (29%)	0.813
LV GLS (%)	−20.9 ± 3.7	−19.4 ± 2.5	0.283
MAPSE (mm)	19 ± 3	20 ± 4	0.673
**CMR VARIABLES**			
RV apex base diameter (mm)	80 ± 13	81 ± 11	0.313
RV mid diameter (mm)	45 ± 7	45 ± 6	0.779
RV basal diameter (mm)	54 ± 7	56 ± 5	0.155
RV free wall thickness (mm)	6 ± 2	5 ± 1	0.591
RV end diastolic volume (mL)	204 ± 48	200 ± 48	0.295
RVEF (%)	39 ± 6	40 ± 6	0.788
RV stroke volume (mL)	79 ± 16	80 ± 18	0.430
LVEF (%)	56 ± 6	59 ± 9	0.396
LV stroke volume (mL)	83 ± 26	76 ± 20	0.308

* significant p-value;

a *hemodynamically significant lesion at t = 1 or t = 2; bpm, beats per minute; BSA, body surface area; ccTGA, congenitally corrected transposition of the great arteries; CMR, cardiac magnetic resonance imaging; FAC, fractional area change; GLS, global longitudinal strain; LV, left ventricle; LVEF, left ventricular ejection fraction; LVOT, left ventricular outflow tract; MAPSE, mitral annular plane systolic excursion; NYHA, New York Heart Association; RV, right ventricle; RVEF, right ventricular ejection fraction; SD, standard deviation; TAPSE, tricuspid annular plane systolic excursion; TGA, transposition of the great arteries; TVP, tricuspid valve annuloplasty; TVR, tricuspid valve replacement; VSD, ventricular septal defect*.

### Cardiac Function at *T* = 1 and *T* = 2

Overall, there were few significant changes in imaging variables between the first and second time points; only the echocardiographic RV apex-base diameter increased significantly (Table 2) (the CMR-derived apex-base diameter did not). To provide context for the values and aid in the interpretation, Table 1 shows reference values for the variables given as published in 12 previous, frequently cited imaging studies in patients with systemic RV (Table 1).

### Correlations Between Echocardiographic and CMR Variables

**Figure 2:**
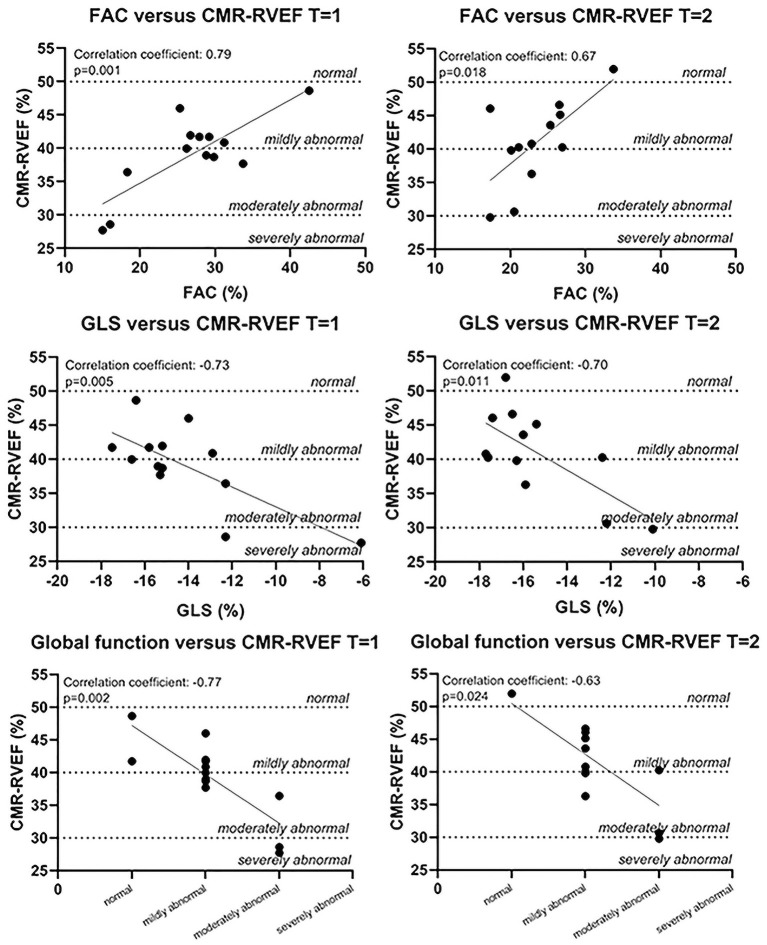
Correlations between echocardiographic variables and CMR-RVEF. Normal values for MRI were based on reference values for the subpulmonary RV (32). CMR-RVEF, cardiac magnetic resonance imaging-derived (systemic) right ventricular ejection fraction; FAC, fractional area change; GLS, global longitudinal strain.

**Figure 3:**
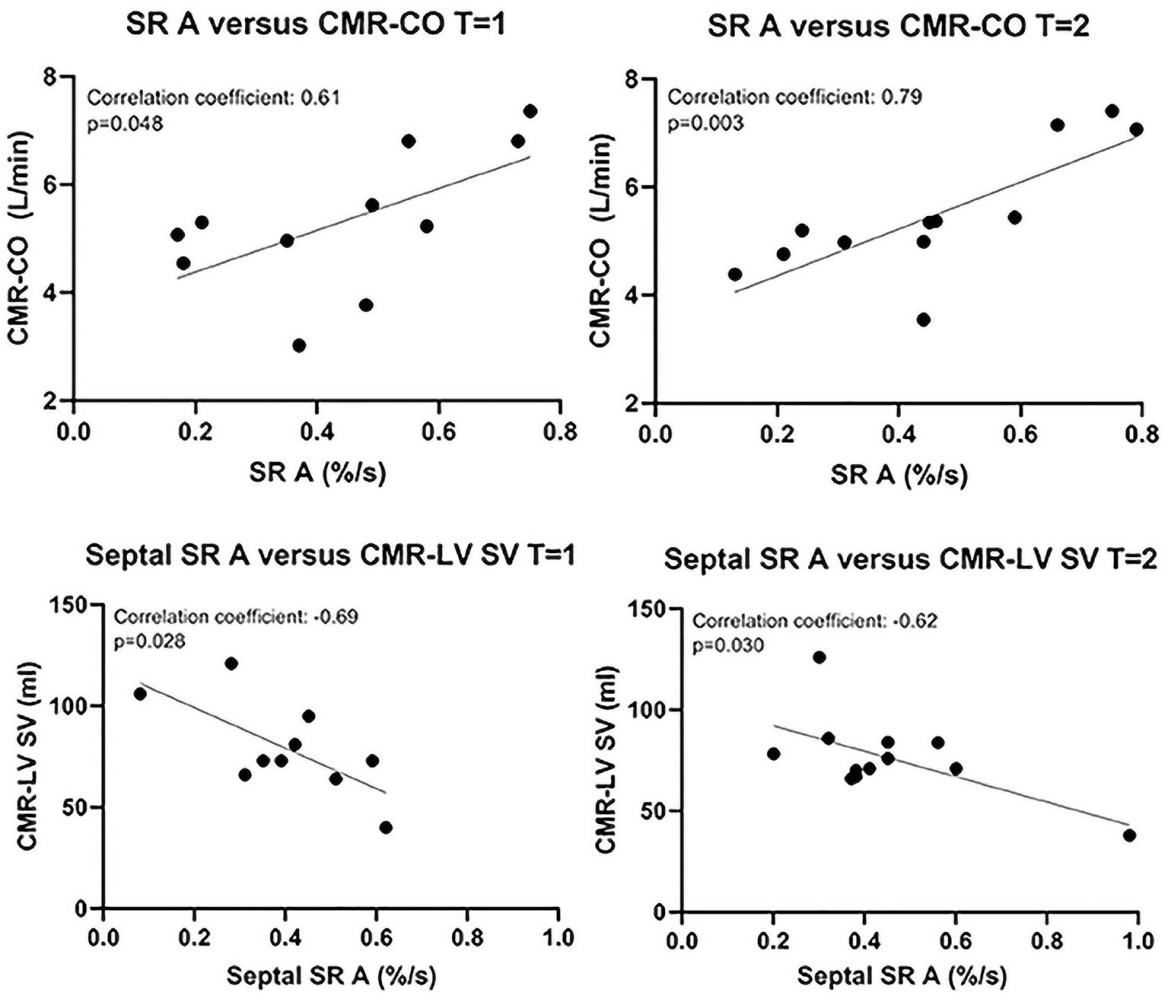
Correlations between echocardiographic and CMR variables other than RVEF. CMR-CO, cardiac magnetic resonance imaging-derived cardiac output; CMR-LV SV, cardiac magnetic imaging-derived (subpulmonary) left ventricular stroke volume; SR A, maximal late diastolic strain rate.

Only three echocardiographic variables of systemic RV function were consistently correlated with CMR-RVEF: visually assessed global systemic RV function (*T* = 1: *r* = −0.77 and *p* = 0.002; *T* = 2: *r* = −0.63 and *p* = 0.024), FAC (*T* = 1: *r* = 0.79 and *p* = 0.001; *T* = 2: *r* = 0.67 and *p* = 0.018), and GLS (*T* = 1: *r* = −0.73 and *p* = 0.005; *T* = 2: *r* = −0.70 and *p* = 0.011) (Figure 2). The late global diastolic SR of the systemic RV (SR A) was consistently significantly correlated with CMR-derived cardiac output. Septal SR A was consistently significantly correlated with LV SV (Figure 3). All other echocardiographic variables were not (consistently) significantly correlated with CMR variables (Supplementary Tables 1–8). As the variables describing atrial dimensions (Table 1) were less clinically relevant in the patients who underwent atrial switch (the majority) and, thus, had surgically altered atria, these variables were not analyzed further.

As SR A and septal SR A (global and septal strain rate during late diastole, respectively) might be heart rate–dependent, analysis was repeated with correction for heart rate (using the partial correlations command in SPSS). After this correction, the correlations between SR A and RV CO and between septal SR A and LV SV were no longer significant. The correlation between FAC and RVEF was still apparent at *T* = 1 and just under the level of significance at *T* = 2. The correlation between GLS and RVEF was not altered by correction for heart rate (Supplementary Table 9).

Of note, regarding systemic RV dimensions, the echocardiographic apex-base diameter showed consistent significant agreement with the CMR-derived apex-base diameter (ICC = 0.74 and *p* = 0.002 at *T* = 1; ICC = 0.62 and *p* = 0.009 at *T* = 2). The other dimensions (mid-diameter, base diameter, and wall thickness) did not show consistent significant agreement between echocardiographic and CMR measurements (Supplementary Table 10).

### The Influence of Tricuspid Regurgitation

To address the possible influence of tricuspid regurgitation (TR) on the assessment of systemic RV function, correlations were calculated between the degree of TR, global RV function, FAC, and GLS, and CMR-RVEF (Supplementary Table 11). No significant correlations were found.

### Interobserver Agreement

**Figure 4:**
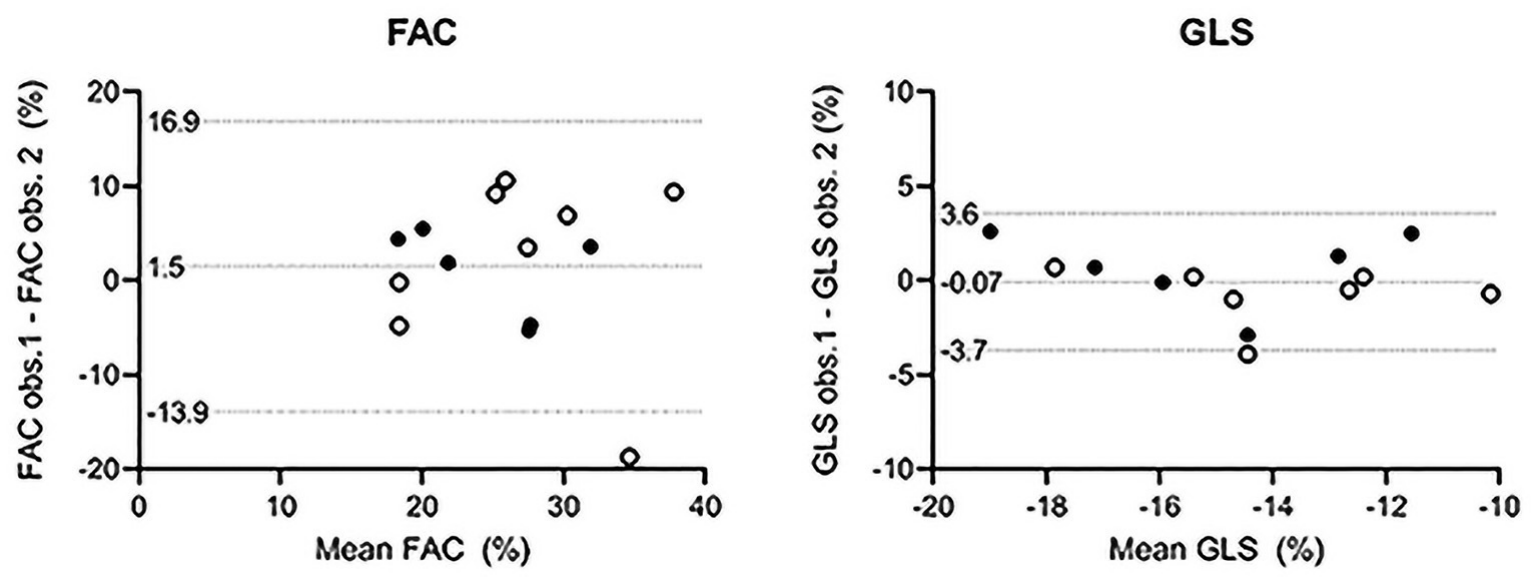
Bland–Altman plots. Open circles: *T* = 1. Filled circles: *T* = 2. The differences between the two observers are plotted on the *y*-axis and the mean value of the variable on the *x*-axis. The mean differences of all observations are close to zero in both cases, indicating no important bias between the two observers. No formal conclusion can be drawn from the constructed limits of agreement because measurements from the same patients at both time points are included. FAC, fractional area change; GLS, global longitudinal strain; obs., observer.

The ICC for FAC is poor at *T* = 1 (ICC = 0.35, *p* = 0.196) and moderate at *T* = 2 (ICC = 0.70, *p* = 0.051). For GLS, the ICC is good at both time points (ICC = 0.82, *p* = 0.006, and ICC = 0.77, *p* = 0.024, respectively). The measurements of both observers are visualized in Bland–Altman plots (Figure 4). This visually confirms that the limits of agreement (dotted lines) of GLS seem acceptable although the limits of agreement of FAC seem moderately large.

Interobserver agreement was also determined for each of the different ways of calculating the MPI. The MPI calculated from the TR curve and the RVOT signal was most reliable with ICC coefficients of 0.84 (*p* = 0.001) and 0.91 (*p* = 0.001), respectively. The other methods were considerably less reliable. None of the methods consistently correlated with CMR-RVEF, CO, or SV (see Supplementary Material).

### Feasibility

From most echocardiograms, FAC (96%), GLS (96%), and (septal) SR A (both 93%) were successfully obtained. For RV dP/dt and LV end-diastolic diameter from the parasternal long axis image, the image quality was often insufficient. The older echocardiograms did not contain pulsed-wave TDI images, but measurements were possible in 10 out of the 14 echocardiograms at *T* = 2.

In addition, CMR-RVEF could not be determined in one patient at *T* = 1 and in one other patient at *T* = 2 because the raw images were no longer available.

## Discussion

### Key Findings and Context Within Literature

**Figure 5:**
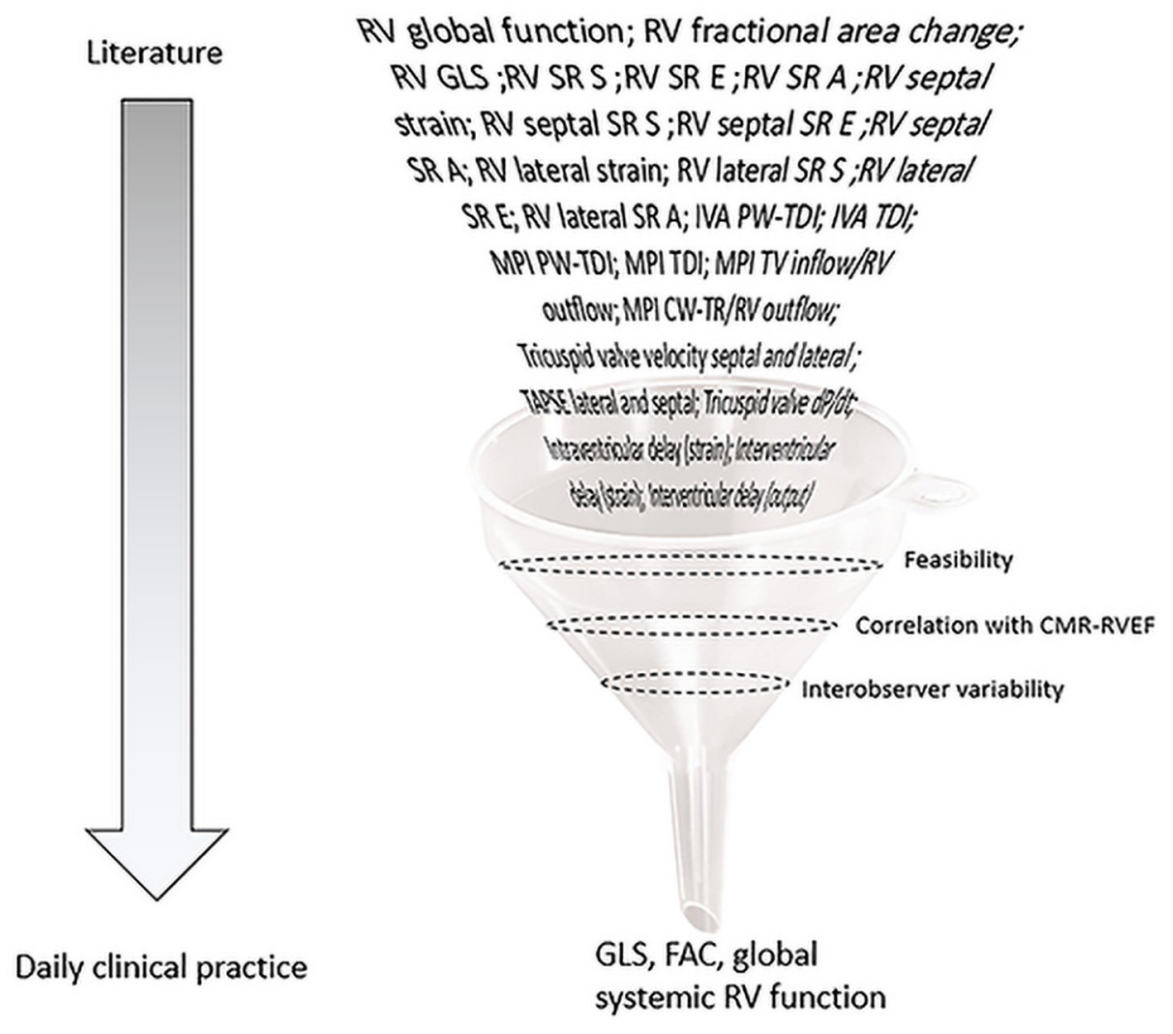
Graphical representation of selection of echocardiographic variables abstracted from literature to assess systemic RV function. FAC, fractional area change; GLS, global longitudinal strain; IVA, isovolumic acceleration; LV, left ventricle; MAPSE, mitral annular plane systolic excursion; MPI, myocardial performance index; PLAX, parasternal long-axis view; PW-TDI, pulsed-wave tissue doppler imaging; RV, right ventricle; SR A, maximal late diastolic strain rate; SR E, maximal early diastolic strain rate; SR S, maximal systolic strain rate; TAPSE, tricuspid annular plane systolic excursion.

We aimed to identify reproducible echocardiographic variables that consistently correlated, i.e., at two points in time, with the gold standard of CMR-RVEF in a cohort of patients with systemic RV from daily clinical practice. The key findings are (Figure 5) that, in this cohort of patients with a systemic RV, (1) FAC, GLS, and global systemic RV function are consistently correlated with CMR-RVEF; (2) GLS shows a good interobserver agreement although the agreement is lower for FAC; (3) measurement of FAC and GLS is feasible in most cases (96%); and (4) other echocardiographic variables, including the MPI, IVA, TAPSE, and s' were not consistently correlated with CMR-RVEF.

To our knowledge, this is the first study to take a range of this extent of echocardiographic measurements of systemic RV function into account and to assess these for consistent correlation over two points in time. From these variables, FAC and especially GLS appear to be most useful. This is in line with previous work: Other studies consistently describe significant correlations between FAC and CMR-RVEF and GLS and CMR-RVEF although correlations between other echocardiographic variables and CMR-RVEF are less robust (11, 12, 14). Previous work also highlights the clinical relevance of FAC and GLS in patients with systemic RV. Lower FAC and higher (worse) GLS were associated with high-sensitive troponin T, a heart failure marker (33). Worse GLS was further associated with lower VO_2max_, heart failure, higher NYHA class, ventricular arrhythmias, and mortality (11, 12, 14, 15, 18, 21).

Estimation of the global RV function by visual assessment also correlated well with CMR. Although it is imprecise, it allows for an adequate first impression. Given the challenging echocardiographic windows in patients with a systemic RV, there is still is a place for visual assessment of the global systemic RV function in cases that do not allow quantification.

Measurement of GLS was more reliable than measurement of FAC. This is to be expected as it is difficult to exactly delineate the endocardial border of the systemic RV: Complex geometry, difficult delineation of the free wall, and the presence of trabeculations and a pronounced moderator band all contribute to this. Slight differences in delineation of the endocardial border between observers may lead to considerable differences in the calculated FAC. Previous studies demonstrate good interobserver agreement for GLS (11, 34) and fair to good agreement for FAC (35, 36). However, the interobserver agreement of FAC has not often been studied in systemic RV but rather in subpulmonary RV, which is easier to delineate. The strength of GLS is that the software shows how the drawn speckles move along with the respective myocardial segments during the cardiac cycle, and discrepancies can be adjusted before definitive calculation. In the case of FAC, checking whether the drawn end-diastolic outline and end-systolic outline actually follow the same contour is less straightforward.

We found no consistent significant correlations between CMR-RVEF and any type of MPI (also called TEI index). Previous studies show conflicting results (6, 9, 10, 16, 37). The reliability of the MPI is limited for each of its possible calculations: when calculated from the TR/pulsed wave signal of TV inflow and the doppler signal of the RVOT, a possible error is introduced due to a difference in heart rate between the two different images. When calculated from (pulsed wave)-TDI images, in which it may be difficult to define the intervals, the measurement is imprecise.

We also found no consistent significant correlations between CMR-RVEF and TAPSE, neither lateral nor septal. Previous studies show conflicting results (10, 19). TAPSE provides limited information because it measures local myocardial function. In the systemic RV, circumferential shortening may contribute more than longitudinal shortening compared with the subpulmonary RV, reducing the ability of TAPSE to reflect global systemic RV function (11). The same limitation may apply to s', which also shows no consistent correlation with RVEF.

Myocardial dyssynchrony is an important problem in patients with systemic RV, especially in patients receiving univentricular pacing (38). Resynchronization therapy is increasingly and successfully used (23, 30). Following previously published methodology, we calculated an intraventricular and an interventricular delay with measurements of the time to peak longitudinal strain in the RV free wall, the septum, and the LV free wall and calculated an interventricular delay from the times to start of output in the doppler signals of the RVOT and LVOT (Supplementary Table 7) (16, 19, 29, 30). Only one of these studies assessed the relation between these measures of dyssynchrony and CMR-RVEF and found a significant negative correlation (19). In the present study, however, we found no consistent correlations with CMR-RVEF. Possible explanations for this include that too few patients with considerable dyssynchrony were included in this cohort as few patients had a QRS duration>130 ms, that the optimal echocardiographic variable to identify dyssynchrony in patients with a systemic RV has yet to be identified, or that RVEF lacks correlation with dyssynchrony and can overestimate cardiac output in cases of dyssynchrony. Future studies correlating echocardiographic measures of dyssynchrony with invasive measurement of cardiac output as well as CMR-RVEF would be valuable.

The current data show no consistent correlation between dP/dt and CMR-RVEF. DP/dt reflects contractility: it assesses the time the systemic RV takes for the build-up of a certain level of pressure during isovolumic contraction and is, therefore, afterload independent. However, its measurement is highly subject to variation, especially with higher values.

Surprisingly, we found that SR A (late diastolic SR) was consistently positively correlated with CMR-cardiac output, and septal SR A was consistently negatively correlated with CMR-LV stroke volume. However, both of these correlations disappeared after correction for heart rate, possibly because a higher heart rate shortens diastolic filling time and generally increases diastolic strain rate. This may imply that strain rate measurements need to be corrected for heart rate.

In patients with a systemic RV, TR is a common consequence of systemic RV annulus dilation (39) and may lead to overestimation of systemic RV function. However, the present results do not show significant correlations between TR and the echocardiographic variables correlating with CMR-RVEF. Previous work in patients with a systemic RV additionally shows that TR was not correlated with CMR-RVEF (40). Although TR may also lead to overestimated systemic RV function as measured by CMR-RVEF, CMR-RVEF correlates well with clinical events in patients with a systemic RV (4, 5), and therefore, can still be used to assess systemic RV function regardless of TR.

Of note, although in this study no functional echocardiographic variables changed significantly over time, the RV apex-base diameter was significantly larger at *T* = 2. Also, the CMR and echocardiographic apex-base measurements show good agreement. This might indicate that RV lengthening may be a sensitive parameter of deterioration, which may be visible before the functional variables significantly deteriorate. This needs to be confirmed in larger studies.

### Study Limitations

A considerable part of our cohort of patients with systemic RV did not meet the inclusion criteria regarding the availability of CMR and echocardiographic images, reflecting routine clinical practice but also limiting the sample size and introducing a possible selection bias causing patients with intracardiac devices to potentially be underrepresented. Furthermore, some correlation coefficients are based on a small sample size as their measurement was not feasible in a considerable number of cases. However, this reflects the practical applicability in routine clinical practice. Also, the interpretation of the RV wall thickness in the context of RV hypertrophy is limited as this requires short axis cine images unavailable in all but one patient. The measurements given were performed in four-chamber views, which is an inferior alternative. Last, the applicability of GLS is limited by variability introduced by spatial and temporal smoothing algorithms (although this affects segmental strain more than global strain, which we used) and by intervendor agreement (41). In this study, all measurements were performed in Echopac by GE Medical Systems.

## Conclusion and Clinical Implications

In conclusion, from all echocardiographic variables available from literature, GLS appears to be the most robust variable to quantify systemic RV function over time. FAC can also be used well-provided that the endocardial border is traced in a consistent manner over time. In case no quantification of the systemic RV function can be made, visual assessment is an adequate substitute (Figure 5).

## Data Availability Statement

The raw data supporting the conclusions of this article will be made available by the authors, upon reasonable request.

## Ethics Statement

The studies involving human participants were reviewed and approved by Medical Research Ethics Committee Leiden University Medical Center. Written informed consent for participation was not required for this study in accordance with the national legislation and the institutional requirements.

## Author Contributions

TZ aided in the design of the study, performed echocardiographic measurements, analyzed the data, and drafted and adjusted the manuscript. MJ aided in the design of the study, the interpretation of the data, guided the drafting of the manuscript, and critically revised and approved the final manuscript. RW provided input for the design of the study, performed MRI measurements, aided in the interpretation of the data, and critically revised and approved the final manuscript. AH provided input for the design of the study, trained TZ to perform echocardiographic measurements, and critically revised and approved the final manuscript. EH provided input for the design of the study, trained TZ to perform echocardiographic measurements, and critically revised and approved the final manuscript. BM guided the drafting of the statistical analysis plan, critically revised its execution and interpretation, and critically revised and approved the final manuscript. HV aided in the design of the study, the interpretation of the data, and critically revised and approved the final manuscript. AE aided in the design of the study, the interpretation of the data, guided the drafting of the manuscript, and critically revised and approved the final manuscript. MS critically revised and approved the final manuscript. PK conceived the study, trained TZ to perform echocardiographic measurements, performed echocardiographic measurements, aided in interpretation of the data, guided the drafting of the manuscript, and critically revised and approved the final manuscript. All authors contributed to the article and approved the submitted version.

## Conflict of Interest

The authors declare that the research was conducted in the absence of any commercial or financial relationships that could be construed as a potential conflict of interest.

## References

[1] Vejlstrup N Sorensen K Mattsson E Thilen U Kvidal P Johansson B. Long-term outcome of mustard/senning correction for transposition of the great arteries in Sweden and Denmark. Circulation. (2015) 132:633–8. 10.1161/CIRCULATIONAHA.114.010770 26185211

[2] Filippov AA Del Nido PJ Vasilyev NV. Management of systemic right ventricular failure in patients with congenitally corrected transposition of the great arteries. Circulation. (2016) 134:1293–302. 10.1161/CIRCULATIONAHA.116.022106 27777298

[3] Iriart X Roubertie F Jalal Z Thambo J-B. Quantification of systemic right ventricle by echocardiography. Arch Cardiovasc Dis. (2016) 109:120–7. 10.1016/j.acvd.2015.11.008 26850171

[4] Helbing WA Rebergen SA Maliepaard C Hansen B Ottenkamp J Reiber JH. Quantification of right ventricular function with magnetic resonance imaging in children with normal hearts and with congenital heart disease. Am Heart J. (1995) 130:828–37. 10.1016/0002-8703(95)90084-5 7572593

[5] Winter MM Bernink FJ Groenink M Bouma BJ Van Dijk AP Helbing WA. Evaluating the systemic right ventricle by CMR: the importance of consistent and reproducible delineation of the cavity. J Cardiovasc Magn Reson. (2008) 10:40. 10.1186/1532-429X-10-40 18713464 PMC2533306

[6] Salehian O Schwerzmann M Merchant N Webb GD Siu SC Therrien J. Assessment of systemic right ventricular function in patients with transposition of the great arteries using the myocardial performance index: comparison with cardiac magnetic resonance imaging. Circulation. (2004) 110:3229–33. 10.1161/01.CIR.0000147284.54140.73 15533860

[7] Rentzsch A Abd El Rahman MY Hui W Helweg A Ewert P Gutberlet M. Assessment of myocardial function of the systemic right ventricle in patients with D-transposition of the great arteries after atrial switch operation by tissue Doppler echocardiography. Z Kardiol. (2005) 94:524–31. 10.1007/s00392-005-0258-6 16049654

[8] Winter MM Bouma BJ Hardziyenka M De Bruin-Bon RH Tan HL Konings TC. Echocardiographic determinants of the clinical condition in patients with a systemic right ventricle. Echocardiography. (2010) 27:1247–55. 10.1111/j.1540-8175.2010.01233.x 20584069

[9] Khattab K Schmidheiny P Wustmann K Wahl A Seiler C Schwerzmann M. Echocardiogram versus cardiac magnetic resonance imaging for assessing systolic function of subaortic right ventricle in adults with complete transposition of great arteries and previous atrial switch operation. Am J Cardiol. (2013) 111:908–13. 10.1016/j.amjcard.2012.11.044 23276471

[10] De Caro E Bondanza S Calevo MG Trocchio G Lupi G Domenicucci S. Tricuspid annular plane systolic excursion for the assessment of ventricular function in adults operated on with mustard procedure for complete transposition of the great arteries. Congenit Heart Dis. (2014) 9:252–8. 10.1111/chd.12135 24010728

[11] Eindhoven JA Menting ME Van Den Bosch AE Mcghie JS Witsenburg M Cuypers JA. Quantitative assessment of systolic right ventricular function using myocardial deformation in patients with a systemic right ventricle. Eur Heart J Cardiovasc Imaging. (2015) 16:380–8. 10.1093/ehjci/jeu194 25300523

[12] Lipczynska M Szymanski P Kumor M Klisiewicz A Mazurkiewicz L Hoffman P. Global longitudinal strain may identify preserved systolic function of the systemic right ventricle. Can J Cardiol. (2015) 31:760–6. 10.1016/j.cjca.2015.02.028 25935885

[13] Shafer KM Mann N Hehn R Ubeda Tikkanen A Valente AM Geva T. Relationship between exercise parameters and noninvasive indices of right ventricular function in patients with biventricular circulation and systemic right ventricle. Congenit Heart Dis. (2015) 10:457–65. 10.1111/chd.12248 25597937 PMC4506894

[14] Kowalik E Mazurkiewicz L Kowalski M Klisiewicz A Marczak M Hoffman P. *Echocardiography* vs magnetic resonance imaging in assessing ventricular function and systemic atrioventricular valve status in adults with congenitally corrected transposition of the great arteries. Echocardiography. (2016) 33:1697–702. 10.1111/echo.13339 27542349

[15] Ladouceur M Redheuil A Soulat G Delclaux C Azizi M Patel M. Longitudinal strain of systemic right ventricle correlates with exercise capacity in adult with transposition of the great arteries after atrial switch. Int J Cardiol. (2016) 217:28–34. 10.1016/j.ijcard.2016.04.166 27179205

[16] Iriart X Horovitz A Van Geldorp IE Barnetche T Lederlin M De Guillebon M. The role of echocardiography in the assessment of right ventricular systolic function in patients with transposition of the great arteries and atrial redirection. Arch Cardiovasc Dis. (2012) 105:432–41. 10.1016/j.acvd.2012.05.005 22958886

[17] Ruotsalainen HK Bellsham-Revell HR Bell AJ Pihkala JI Ojala TH Simpson JM. Right ventricular systolic function in hypoplastic left heart syndrome: a comparison of manual and automated software to measure fractional area change. Echocardiography. (2017) 34:587–93. 10.1111/echo.13470 28191731

[18] Kalogeropoulos AP Deka A Border W Pernetz MA Georgiopoulou VV Kiani J. Right ventricular function with standard and speckle-tracking echocardiography and clinical events in adults with D-transposition of the great arteries post atrial switch. J Am Soc Echocardiogr. (2012) 25:304–12. 10.1016/j.echo.2011.12.003 22196884

[19] Chow PC Liang XC Lam WW Cheung EW Wong KT Cheung YF. Mechanical right ventricular dyssynchrony in patients after atrial switch operation for transposition of the great arteries. Am J Cardiol. (2008) 101:874–81. 10.1016/j.amjcard.2007.11.033 18328857

[20] Helsen F De Meester P Van De Bruaene A Gabriels C Santens B Claeys M. Right ventricular systolic dysfunction at rest is not related to decreased exercise capacity in patients with a systemic right ventricle. Int J Cardiol. (2018) 260:66–71. 10.1016/j.ijcard.2018.03.029 29530621

[21] Diller GP Radojevic J Kempny A Alonso-Gonzalez R Emmanouil L Orwat S. Systemic right ventricular longitudinal strain is reduced in adults with transposition of the great arteries, relates to subpulmonary ventricular function, and predicts adverse clinical outcome. Am Heart J. (2012) 163:859–66. 10.1016/j.ahj.2012.01.038 22607865

[22] Lancellotti P Moura L Pierard LA Agricola E Popescu BA Tribouilloy C. European Association of Echocardiography recommendations for the assessment of valvular regurgitation. Part 2: mitral and tricuspid regurgitation (native valve disease). Eur J Echocardiogr. (2010) 11:307–32. 10.1093/ejechocard/jeq031 20435783

[23] Janousek J Tomek V Chaloupecky VA Reich O Gebauer RA Kautzner J. Cardiac resynchronization therapy: a novel adjunct to the treatment and prevention of systemic right ventricular failure. J Am Coll Cardiol. (2004) 44:1927–31. 10.1016/j.jacc.2004.08.044 15519030

[24] Rudski LG Lai WW Afilalo J Hua L Handschumacher MD Chandrasekaran K. Guidelines for the echocardiographic assessment of the right heart in adults: a report from the American Society of Echocardiography endorsed by the European Association of Echocardiography, a registered branch of the European Society of Cardiology, and the Canadian Society of Echocardiography. J Am Soc Echocardiogr. (2010) 23:685–713. 10.1016/j.echo.2010.05.010 20620859

[25] Zoghbi WA Enriquez-Sarano M Foster E Grayburn PA Kraft CD Levine RA. American Society of Echocardiography: recommendations for evaluation of the severity of native valvular regurgitation with two-dimensional and Doppler echocardiography?: a report from the American Society of Echocardiography's Nomenclature and Standards Committee and The Task Force on Valvular Regurgitation, developed in conjunction with the American College of Cardiology Echocardiography Committee, The Cardiac Imaging Committee, Council on Clinical Cardiology, The American Heart Association, and the European Society of Cardiology Working Group on Echocardiography, represented by. Eur Heart J. (2003) 4:237–61. 10.1016/j.euje.2003.07.001 12835667

[26] Tei C Ling LH Hodge DO Bailey KR Oh JK Rodeheffer RJ. New index of combined systolic and diastolic myocardial performance: a simple and reproducible measure of cardiac function–a study in normals and dilated cardiomyopathy. J Cardiol. (1995) 26:357–66. 10.1016/S0894-7317(05)80111-7 8558414

[27] Olson JM Samad BA Alam M. Myocardial performance index determined by tissue doppler imaging in patients with systolic heart failure predicts poor long-term prognosis: an observational cohort study. J Card Fail. (2016) 22:611–7. 10.1016/j.cardfail.2016.01.005 26777759

[28] Vogel M Schmidt MR Kristiansen SB Cheung M White PA Sorensen K. Validation of myocardial acceleration during isovolumic contraction as a novel noninvasive index of right ventricular contractility: comparison with ventricular pressure-volume relations in an animal model. Circulation. (2002) 105:1693–9. 10.1161/01.CIR.0000013773.67850.BA 11940549

[29] Forsha D Risum N Smith PB Kanter RJ Samad Z Barker P. Frequent activation delay-induced mechanical dyssynchrony and dysfunction in the systemic right ventricle. J Am Soc Echocardiogr. (2016) 29:1074–83. 10.1016/j.echo.2016.08.002 27624591

[30] Jauvert G Rousseau-Paziaud J Villain E Iserin L Hidden-Lucet F Ladouceur M. Effects of cardiac resynchronization therapy on echocardiographic indices, functional capacity, and clinical outcomes of patients with a systemic right ventricle. Europace. (2009) 11:184–90. 10.1093/europace/eun319 19038975

[31] Altman DG Bland JM. Measurement in medicine: the analysis of method comparison studies. The Statistician. (1983) 32:307–17. 10.2307/2987937

[32] Petersen SE Khanji MY Plein S Lancellotti P Bucciarelli-Ducci C. European Association of Cardiovascular Imaging expert consensus paper: a comprehensive review of cardiovascular magnetic resonance normal values of cardiac chamber size and aortic root in adults and recommendations for grading severity. Eur Heart J. (2019) 20:1321–31. 10.1093/ehjci/jez232 31544926

[33] Abiko M Inai K Shimada E Asagai S Nakanishi T. The prognostic value of high sensitivity cardiac troponin T in patients with congenital heart disease. J Cardiol. (2018) 71:389–93. 10.1016/j.jjcc.2017.09.012 29108668

[34] Chow P-C Liang XC Cheung E Lam W Cheung YF. New two-dimensional global longitudinal strain and strain rate imaging for assessment of systemic right ventricular function. Heart. (2008) 94:855–9. 10.1136/hrt.2007.131862 18230639

[35] Pinedo M Villacorta E Tapia C Arnold R Lopez J Revilla A. Inter- and intra-observer variability in the echocardiographic evaluation of right ventricular function. Rev Esp Cardiol. (2010) 63:802–9. 10.1016/S0300-8932(10)70183-4 20609314

[36] Chaix MA Dore A Marcotte F Shohoudi A Labombarda F Mercier LA. Variability in the echocardiographic evaluation of the systemic right ventricle. Can J Cardiol. (2019) 35:178–84. 10.1016/j.cjca.2018.11.021 30760424

[37] Lissin LW Li W Murphy DJJr Hornung T Swan L Mullen M. Comparison of transthoracic echocardiography versus cardiovascular magnetic resonance imaging for the assessment of ventricular function in adults after atrial switch procedures for complete transposition of the great arteries. Am J Cardiol. (2004) 93:654–7. 10.1016/j.amjcard.2003.11.044 14996604

[38] Horovitz A De Guillebon M Van Geldorp IE Bordachar P Roubertie F Iriart X. Effects of nonsystemic ventricular pacing in patients with transposition of the great arteries and atrial redirection. J Cardiovasc Electrophysiol. (2012) 23:766–70. 10.1111/j.1540-8167.2011.02271.x 22429270

[39] Brida M Diller G-P Gatzoulis MA. Systemic right ventricle in adults with congenital heart disease. Circulation. (2018) 137:508–18. 10.1161/CIRCULATIONAHA.117.031544 29378757

[40] Lewis M Ginns J Rosenbaum M. Is systemic right ventricular function by cardiac MRI related to the degree of tricuspid regurgitation in congenitally corrected transposition of the great arteries? Int J Cardiol. (2014) 174:586–9. 10.1016/j.ijcard.2014.04.129 24814545

[41] Amzulescu MS De Craene M Langet H Pasquet A Vancraeynest D Pouleur AC. Myocardial strain imaging: review of general principles, validation, and sources of discrepancies. Eur Heart J. (2019) 20:605–19. 10.1093/ehjci/jez041 30903139 PMC6529912

