# Supplementary material for: Validation and Feasibility of Echocardiographic Assessment of Systemic Right Ventricular Function: Serial Correlation With MRI
Source: Front Cardiovasc Med. 2021 Mar 16;8:644193. doi: 10.3389/fcvm.2021.644193 (PMC8008818; doi:10.3389/fcvm.2021.644193)
Supplement: Supplementary file 1 [file Data_Sheet_1.docx]

**Supplementary material**

**Supplementary figure 1: methods to calculate the myocardial performance index**


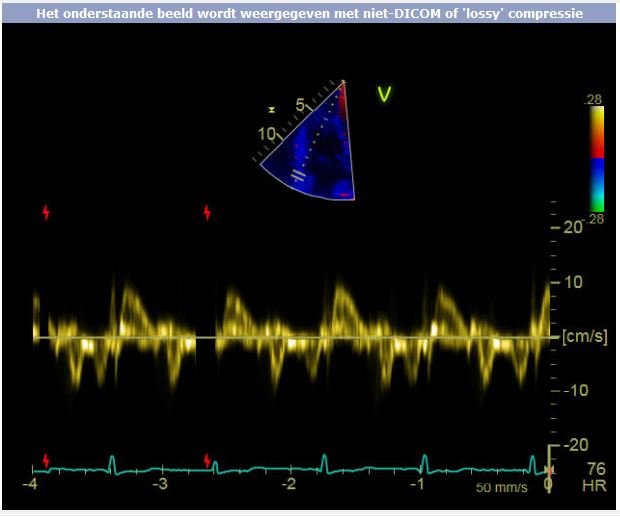

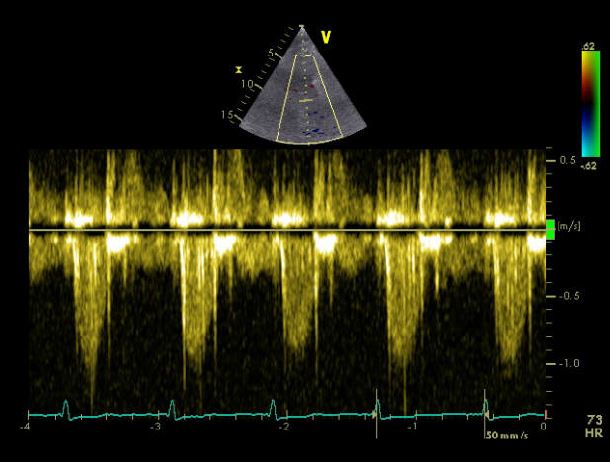

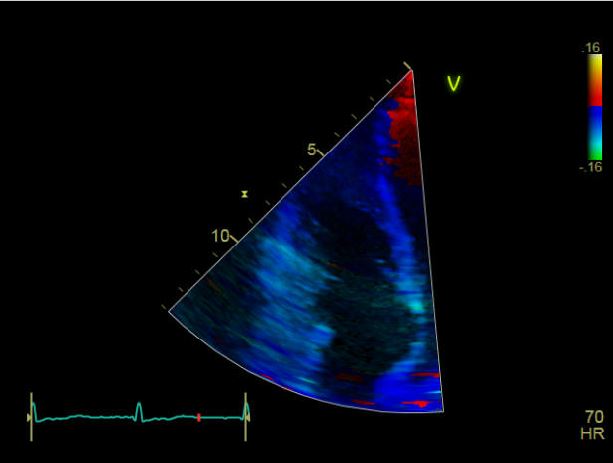

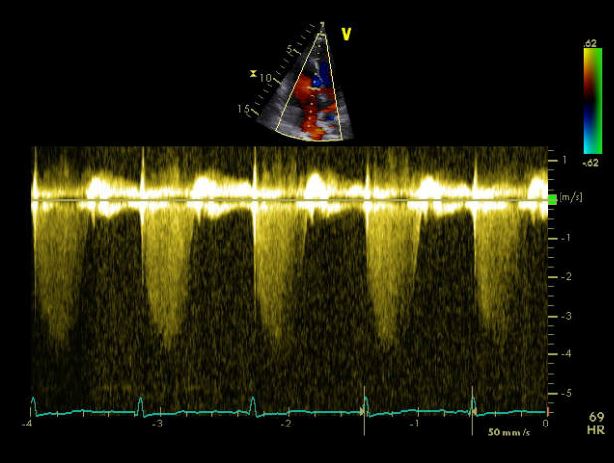

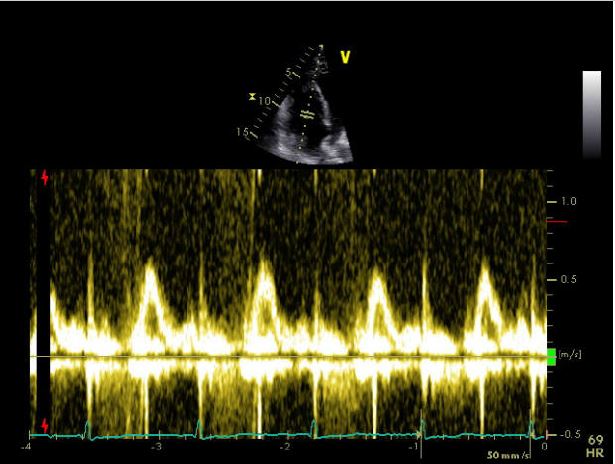


ET+ICT+IRT

ET

ET+ICT+IRT

ET+ICT+IRT

ET

**A**

**B**

**C**

**D**

**E**

Calculation of the myocardial performance index (MPI): (isovolumic contraction time+isovolumic relaxation time)/ejection time. A: pulsed-wave tissue doppler image of tricuspid valve in apical four chamber view allows measurement of the ejection time (ET) and the total of the isovolumic contraction (ICT)+ET+isovolumic relaxation (IRT). B: similarly to A, from the tissue doppler image, the ET and ICT+ET+IRT can be measured when the caliper is placed at the lateral aspect of the tricuspid valve. C: the duration of the tricuspid regurgitation signal equals ICT+ET+IRT. Combined with the ET in E, the MPI can be calculated. D: the duration between the end of the A-wave and the beginning of the E-wave in the pulsed wave (or continuous wave) tricuspid valve inflow signal equals ICT+ET+IRT. Combined with the ET in E, the MPI can be calculated. E: the duration of the doppler signal of the right ventricular outflow tract=ET.

**Supplementary table 1: correlations between classic echocardiographic variables and CMR variables at T=1**

|  |  | RVEF | RVEF_ BSA | CO | CO_BSA | SV | SV_BSA |
| --- | --- | --- | --- | --- | --- | --- | --- |
| FAC | r  P | **0.79**  **0.001** | **0.80**  **0.002** | 0.046  0.886 | 0.222  0.487 | 0.312  0.300 | 0.470  0.123 |
| TAPSE  lateral | r  P | **0.675**  **0.011** | **0.652**  **0.022** | 0.162  0.614 | 0.275  0.386 | 0.373  0.209 | 0.350  0.264 |
| TAPSE septal | r  P | 0.332  0.267 | 0.388  0.213 | -0.369  0.238 | -0.324  0.305 | -0.053  0.864 | 0.200  0.534 |
| S’ lateral | r  P | 0.407  0.278 | 0.461  0.211 | 0.034  0.931 | 0.137  0.725 | -0.095  0.808 | -0.003  0.995 |
| S’ septal | r  P | 0.077  0.833 | 0.126  0.729 | -0.328  0.355 | -0.342  0.333 | 0.190  0.599 | 0.302  0.397 |
| TR grade** | rho  P | 0.281  0.353 | 0.089  0.782 | **0.614**  **0.034** | **0.816**  **0.001** | 0.044  0.886 | -0.063  0.845 |
| SRV global** | rho  P | **-0.774**  **0.002** | **-0.706**  **0.010** | -0.207  0.518 | -0.382  0.220 | -0.2941  0.335 | -0.426  0.168 |

** spearman’s correlation coefficient; _BSA: normalised to body surface area; CO: cardiac output; FAC: fractional area change; RVEF: (systemic) right ventricular ejection fraction; SRV global: global systemic right ventricular function (visual assessment); SV; stroke volume; TAPSE: tricuspid annular plane systolic excursion; TR: tricuspid regurgitation

**Supplementary table 2: correlations between classic echocardiographic variables and CMR variables at T=2**

|  |  | RVEF | RVEF_BSA | CO | CO_BSA | SV | SV_BSA |
| --- | --- | --- | --- | --- | --- | --- | --- |
| FAC | r  P | **0.667**  **0.018** | **0.607**  **0.037** | **0.588**  **0.044** | **0.678**  **0.015** | 0.315  0.318 | 0.364  0.244 |
| TAPSE  lateral | r  P | -0.096  0.766 | -0.165  0.609 | -0.186  0.563 | -0.282  0.374 | -0.082  0.799 | -0.193  0.549 |
| TAPSE septal | r  P | 0.084  0.784 | 0.163  0.594 | -0.115  0.709 | -0.049  0.873 | 0.547  0.053 | **0.623**  **0.023** |
| S’ lateral (pwtdi) | r  P | 0.422  0.258 | 0.459  0.214 | -0.051  0.897 | 0.043  0.913 | -0.187  0.629 | -0.097  0.804 |
| S’ septal (pwtdi) | r  P | -0.056  0.895 | -0.110  0.795 | -0.038  0.928 | -0.121  0.775 | 0.397  0.330 | 0.357  0.385 |
| S’ lateral (tdi) | r  P | 0.373  0.232 | 0.344  0.274 | 0.197  0.540 | 0.244  0.446 | 0.166  0.606 | 0.203  0.527 |
| S’ septal (tdi) | r  P | -0.107  0.728 | -0.093  0.762 | -0.204  0.505 | -0.217  0.477 | -0.008  0.979 | -0.002  0.995 |
| TI grade** | rho  P | 0.300  0.319 | 0.240  0.429 | 0.303  0.314 | 0.373  0.209 | 0.164  0.592 | 0.098  0.750 |
| SRV global** | rho  P | -0.487  0.091 | -0.365  0.220 | **-0.568**  **0.043** | -0.447  0.126 | -0.135  0.659 | 0.014  0.965 |

** spearman’s correlation coefficient; _BSA: normalised to body surface area; CO: cardiac output; FAC: fractional area change; RVEF: (systemic) right ventricular ejection fraction; SRV global: global systemic right ventricular function (visual assessment); SV; stroke volume; TAPSE: tricuspid annular plane systolic excursion; TR: tricuspid regurgitation

**Supplementary table 3: correlations between longitudinal strain variables and CMR variables at T=1**

|  |  | RVEF | RVEF_BSA | CO | CO_BSA | SV | SV_BSA |
| --- | --- | --- | --- | --- | --- | --- | --- |
| GLS | r  P | **-0.726***  **0.005** | **-0.824***  **0.001** | 0.410  0.186 | 0.253  0.428 | -0.215  0.481 | -0.490  0.106 |
| SR S | r  P | **-0.591***  **0.033** | **-0.680***  **0.015** | 0.440  0.152 | 0.324  0.304 | 0.022  0.944 | -0.169  0.599 |
| SR E | r  P | 0.435  0.137 | 0.351  0.263 | 0.527  0.078 | **0.655***  **0.021** | 0.379  0.202 | 0.365  0.243 |
| SR A | r  P | 0.383  0.219 | 0.202  0.551 | **0.607***  **0.048** | 0.592  0.055 | 0.240  0.452 | 0.145  0.671 |
| Septal strain | r  P | -0.165  0.590 | -0.392  0.207 | **0.677***  **0.016** | **0.600***  **0.039** | 0.044  0.886 | -0.269  0.397 |
| Sept SR S | r  P | -0.040  0.898 | -0.277  0.383 | **0.618***  **0.032** | **0.601***  **0.039** | -0.027  0.931 | -0.195  0.543 |
| Septal SR E | r  P | 0.260  0.391 | 0.478  0.116 | 0.216  0.500 | 0.377  0.228 | 0.177  0.563 | 0.265  0.405 |
| Septal SR A | r  P | 0.482  0.113 | 0.148  0.663 | 0.563  0.071 | 0.502  0.115 | -0.062  0.849 | -0.049  0.885 |
| Lateral strain | r  P | -0.137  0.656 | -0.333  0.290 | 0.416  0.178 | 0.334  0.289 | 0.100  0.744 | 0.050  0.876 |
| Lateral SR S | r  P | -0.188  0.538 | -0.232  0.469 | 0.266  0.404 | 0.196  0.541 | 0.119  0.699 | 0.025  0.939 |
| Lateral SR E | r  P | 0.228  0.454 | 0.191  0.553 | 0.292  0.358 | 0.386  0.245 | -0.058  0.851 | -0.024  0.940 |
| Lateral SR A | r  P | 0.104  0.748 | -0.038  0.912 | 0.305  0.361 | 0.288  0.391 | 0.075  0.817 | 0.023  0.947 |

_BSA: normalised to body surface area; CO: cardiac output; RVEF: (systemic) right ventricular ejection fraction; SR A: maximal late diastolic strain rate; SR E: maximal early diastolic strain rate; SR S: maximal systolic strain rate; SV: stroke volume

**Supplementary table 4: correlations between longitudinal strain variables and CMR variables at T=2**

|  |  | RVEF | RVEF_BSA | CO | CO_BSA | SV | SV_BSA |
| --- | --- | --- | --- | --- | --- | --- | --- |
| GLS | r  P | **-0.703**  **0.011** | **-0.662**  **0.019** | -0.513  0.088 | **-0.602**  **0.038** | **-0.591**  **0.043** | **-0.642**  **0.024** |
| SR S | r  P | -0.447  0.145 | -0.558  0.059 | 0.074  0.820 | -0.101  0.755 | 0.249  0.435 | 0.038  0.907 |
| SR E | r  P | 0.494  0.103 | 0.530  0.077 | 0.417  0.177 | 0.542  0.069 | 0.175  0.587 | 0.281  0.377 |
| SR A | r  P | 0.559  0.059 | 0.360  0.251 | **0.785**  **0.003** | **0.727**  **0.007** | 0.127  0.695 | -0.015  0.936 |
| Septal strain | r  P | 0.254  0.425 | 0.061  0.850 | 0.513  0.088 | 0.402  0.195 | -0.320  0.310 | -0.491  0.105 |
| Sept SR S | r  P | 0.257  0.420 | 0.034  0.917 | 0.553  0.062 | 0.408  0.188 | 0.141  0.662 | -0.080  0.805 |
| Sept SR E | r  P | 0.072  0.823 | 0.265  0.404 | -0.359  0.252 | -0.171  0.595 | -0.393  0.206 | -0.123  0.704 |
| Sept SR A | r  P | **0.684**  **0.014** | 0.553  0.062 | **0.785**  **0.002** | **0.815**  **0.001** | 0.300  0.344 | 0.239  0.454 |
| Lateral strain | r  P | **-0.598**  **0.040** | -0.453  0.139 | **-0.645**  **0.024** | **-0.643**  **0.024** | 0.157  0.626 | 0.208  0.516 |
| Lat SR S | r  P | -0.502  0.096 | -0.421  0.173 | -0.349  0.267 | -0.385  0.217 | 0.429  0.164 | 0.415  0.180 |
| Lat SR E | r  P | 0.484  0.111 | 0.437  0.155 | 0.510  0.090 | 0.573  0.051 | -0.176  0.584 | -0.150  0.641 |
| Lat SR A | r  P | 0.220  0.492 | 0.047  0.884 | 0.377  0.227 | 0.279  0.380 | -0.222  0.489 | -0.344  0.274 |

_BSA: normalised to body surface area; CO: cardiac output; RVEF: (systemic) right ventricular ejection fraction; SR A: maximal late diastolic strain rate; SR E: maximal early diastolic strain rate; SR S: maximal systolic strain rate; SV: stroke volume

**Supplementary table 5: correlations between IVA/MPI/dP/dt and CMR variables at T=1**

|  |  | RVEF | RVEF_BSA | CO | CO_BSA | SV | SV_BSA |
| --- | --- | --- | --- | --- | --- | --- | --- |
| IVA TDI | r  P | 0.113  0.772 | 0.305  0.425 | -0.321  0.400 | -0.257  0.504 | -0.218  0.574 | -0.107  0.783 |
| MPI TDI | r  P | -0.265  0.491 | -0.205  0.597 | -0.314  0.410 | -0.328  0.389 | -0.059  0.880 | -0.005  0.990 |
| MPI TV-PW | r  P | -0.364  0.271 | -0.336  0.313 | -0.190  0.576 | -0.292  0.384 | 0.176  0.605 | 0.155  0.649 |
| MPI TR-CW | r  P | -0.560  0.058 | -0.518  0.084 | 0.049  0.879 | -0.036  0.911 | -0.235  0.462 | -0.338  0.283 |
| dP/dt | r  P | 0.335  0.417 | 0.391  0.338 | -0.013  0.975 | 0.090  0.833 | 0.160  0.705 | 0.303  0.466 |

_BSA: normalised to body surface area; CO: cardiac output; IVA: isovolumic acceleration; MPI: myocardial performance index; RVEF: (systemic) right ventricular ejection fraction; SV: stroke volume; TDI: tissue doppler imaging; TR-CW: continuous wave imagine of tricuspid regurgitation; TV-PW: pulsed wave image of tricuspid valve inflow

**Supplementary table 6: correlations between IVA/MPI/dP/dt and CMR variables at T=2**

|  |  | RVEF | RVEF_BSA | CO | CO_BSA | SV | SV_BSA |
| --- | --- | --- | --- | --- | --- | --- | --- |
| IVA PW-TDI | r  P | 0.284  0.459 | 0.376  0.318 | -0.173  0.656 | -0.074  0.849 | -0.140  0.719 | -0.020  0.959 |
| IVA TDI | r  P | **0.597**  **0.040** | **0.641**  **0.025** | 0.161  0.617 | 0.311  0.326 | 0.215  0.503 | 0.368  0.239 |
| MPI PW-TDI | r  P | -0.258  0.502 | -0.081  0.836 | -0.307  0.422 | -0.246  0.523 | 0.627  0.071 | **0.785**  **0.012** |
| MPI TDI | r  P | 0.078  0.809 | -0.032  0.922 | -0.236  0.460 | -0.168  0.601 | **0.612**  **0.035** | **0.710**  **0.010** |
| MPI TV-PW | r  P | 0.078  0.809 | 0.127  0.695 | 0.004  0.991 | 0.051  0.874 | **0.776**  **0.003** | **0.828**  **0.001** |
| MPI TR-CW | r  P | -0.338  0.309 | -0.278  0.408 | -0.399  0.224 | -0.470  0.144 | 0.259  0.443 | 0.207  0.540 |
| dP/dt | r  P | 0.214  0.503 | 0.068  0.834 | 0.379  0.225 | 0.330  0.296 | -0.074  0.820 | -0.127  0.694 |

_BSA: normalised to body surface area; CO: cardiac output; IVA: isovolumic acceleration; MPI: myocardial performance index; RVEF: (systemic) right ventricular ejection fraction; SV: stroke volume; TDI: tissue doppler imaging; TR-CW: continuous wave imagine of tricuspid regurgitation; TV-PW: pulsed wave image of tricuspid valve inflow

**Supplementary table 7: correlations between echocardiographic variables of dyssynchrony, left-sided dimensions/function, diastolic function, and CMR variables at T=1**

|  |  | RVEF | RVEF_BSA | CO | CO_BSA | SV | SV_BSA |
| --- | --- | --- | --- | --- | --- | --- | --- |
| Interventricular delay (strain) | r  P | 0.509  0.075 | 0.424  0.169 | 0.001  0.997 | 0.106  0.743 | -0.076  0.806 | 0.559  0.059 |
| Interventricular delay (outflow) | r  P | -0.308  0.330 | -0.287  0.392 | -0.466  0.149 | -0.559  0.074 | -0.336  0.285 | -0.248  0.461 |
| Intraventricular delay (strain) | r  P | -0.167  0.585 | 0.078  0.808 | -0.299  0.345 | -0.281  0.377 | 0.087  0.778 | 0.154  0.633 |
| LV EDD plax | r  P | **-0.728***  **0.026** | **-0.775***  **0.024** | -0.294  0.479 | -0.406  0.319 | 0.096  0.806 | -0.141  0.738 |
| LV EDD plax - BSA | r  P | **-0.742***  **0.035** | **-0.738***  **0.036** | -0.468  0.242 | -0.538  0.169 | -0.186  0.660 | -0.220  0.600 |
| LV EDD AP4CH | r  P | **-0.628***  **0.022** | **-0.643***  **0.024** | 0.380  0.223 | 0.268  0.399 | 0.332  0.268 | -0.040  0.902 |
| LV EDD AP4CH - BSA | r  P | -0.464  0.129 | -0.396  0.202 | -0.002  0.996 | -0.053  0.870 | -0.028  0.930 | -0.052  0.872 |
| LV GLS | r  P | 0.016  0.962 | -0.040  0.903 | -0.053  0.870 | -0.097  0.763 | 0.081  0.801 | 0.027  0.933 |
| MAPSE | r  P | 0.094  0.771 | 0.215  0.503 | -0.127  0.694 | -0.029  0.928 | -0.104  0.745 | 0.040  0.902 |
| E/A ratio | r  P | -0.096  0.821 | 0.191  0.650 | -0.424  0.296 | -0.321  0.439 | -0.466  0.245 | -0.315  0.447 |
| E/e’ ratio* | r  P | 0.171  0.661 | 0.196  0.613 | -0.221  0.568 | -0.223  0.564 | 0.539  0.134 | 0.644  0.061 |

_BSA: normalised to body surface area; AP4CH: apical four chamber view; CO: cardiac output; GLS: global longitudinal strain; LV EDD; left ventricular end-diastolic diameter; MAPSE: mitral annular plane systolic excursion; Plax: parasternal long axis view; RVEF: (systemic) right ventricular ejection fraction; SV: stroke volume

**Supplementary table 8: correlations between echocardiographic variables of dyssynchrony, left-sided dimensions/function, diastolic function, and CMR variables at T=2**

|  |  | RVEF | RVEF_BSA | CO | CO_BSA | SV | SV_BSA |
| --- | --- | --- | --- | --- | --- | --- | --- |
| Interv. delay (strain) | r  P | 0.005  0.988 | 0.136  0.691 | -0.303  0.364 | -0.183  0.591 | 0.378  0.251 | 0.531  0.093 |
| Interv. delay (outflow) | r  P | -0.301  0.341 | -0.126  0.697 | **-0.628**  **0.029** | -0.565  0.056 | -0.332  0.292 | -0.213  0.506 |
| Intrav. delay (strain) | r  P | 0.510  0.090 | 0.501  .097 | 0.488  0.107 | **0.590****  **0.043** | 0.296  0.349 | 0.375  0.230 |
| LV EDD plax | r  P | -0.449  0.166 | -0.443  0.173 | -0.331  0.320 | -0.408  0.213 | -0.088  0.796 | -0.154  0.651 |
| LV EDD plax - BSA | r  P | -0.295  0.378 | -0.224  0.508 | -0.384  0.243 | -0.362  0.274 | -0.105  0.760 | -0.081  0.814 |
| LV EDD 4CH | r  P | -0.283  0.348 | -0.409  0.165 | 0.204  0.504 | 0.048  0.876 | 0.467  0.108 | 0.302  0.317 |
| LV EDD 4CH - BSA | r  P | -0.211  0.489 | -0.257  0.397 | 0.108  0.726 | 0.035  0.909 | 0.466  0.109 | 0.390  .188 |
| LV GLS | r  P | 0.283  0.373 | 0.320  0.310 | 0.272  0.393 | 0.351  0.263 | 0.048  0.882 | 0.104  0.748 |
| MAPSE | r  P | -0.308  0.306 | -0.386  0.193 | -0.101  0.742 | -0.234  0.442 | 0.210  0.491 | 0.101  0.743 |
| ePAP | r  P | -0.696  0.124 | -0.572  0.236 | -0.642  0.169 | -0.716  0.109 | 0.029  0.956 | 0.050  0.926 |
| E/A ratio | r  P | -0.168  0.602 | -0.044  0.892 | -0.405  0.192 | -0.333  0.291 | -0.304  0.336 | -0.182  0.572 |
| E/e’ ratio* PW-TDI | r  P | -0.385  0.307 | -0.471  0.201 | 0.022  0.955 | -0.132  0.735 | **0.837****  **0.005** | **0.737****  **0.023** |
| E/e’ ratio  TDI | r  P | -0.100  0.756 | -0.050  0.877 | -0.146  0.651 | **-0.128**  **0.693** | **0.642**  **0.024** | **0.677**  **0.016** |

_BSA: normalised to body surface area; AP4CH: apical four chamber view; CO: cardiac output; GLS: global longitudinal strain; LV EDD; left ventricular end-diastolic diameter; MAPSE: mitral annular plane systolic excursion; Plax: parasternal long axis view; RVEF: (systemic) right ventricular ejection fraction; SV: stroke volume

**Supplementary table 9: Correlations between echocardiographic and CMR variables after correction for heart rate**

|  |  | T=1 |  |  | T=2 |  |  |
| --- | --- | --- | --- | --- | --- | --- | --- |
|  |  | RVEF | RV CO | RV SV | RVEF | RV CO | RV SV |
| FAC | r  P | **0.87***  **<0.001** | 0.34  0.303 | 0.31  0.347 | 0.56  0.073 | 0.43  0.184 | 0.50  0.122 |
| GLS | r  P | **-0.81***  **0.002** | -0.32  0.343 | -0.29  0.395 | **-0.77***  **0.005** | **-0.60***  **0.049** | **-0.66***  **0.028** |
| SR A | r  P | 0.27  0.448 | 0.44  0.205 | 0.35  0.322 | 0.23  0.502 | 0.53  0.091 | 0.48  0.137 |
|  |  | T=1 |  |  | T=2 |  |  |
|  |  | LVEF | LV CO | LV SV | LVEF | LV CO | LV SV |
| Septal SR A | r  P | 0.11  0.772 | -0.23  0.556 | -0.30  0.439 | -0.24  0.480 | -0.56  0.074 | -0.41  0.209 |

*: significant p-value; CO: cardiac output; FAC: fractional area change; GLS: global longitudinal strain; LVEF: left ventricular ejection fraction; RVEF: systemic right ventricular ejection fraction; SR A: late diastolic strain rate; SV: stroke volume

**Supplementary table 10: agreement between echocardiographic dimensions and CMR dimensions at T=1 and T=2**

|  |  | RV apex-base | RV mid diameter | RV base diameter | RV free wall |
| --- | --- | --- | --- | --- | --- |
| T=1 | ICC  P | **0.74**  **0.002** | **0.71**  **0.004** | 0.40  0.065 | 0.29  0.088 |
| T=2 | ICC  P | **0.62**  **0.009** | 0.39  0.093 | 0.20  0.14 | -0.16  0.81 |

CMR: cardiac magnetic resonance imaging; ICC: intraclass correlation coefficient; RV: (systemic) right ventricle

**Supplementary table 11: Correlations between tricuspid regurgitation and main variables of systemic RV function**

|  |  | Correlation with TR grade at T=1 | Correlation with TR grade at T=2 |
| --- | --- | --- | --- |
| Echocardiography |  |  |  |
| - Global RV function | rho  p | -0.32  0.26 | -0.38  0.18 |
| - FAC | rho  p | 0.17  0.57 | 0.36  0.23 |
| - GLS | rho  p | -0.32  0.26 | -0.42  0.16 |
| CMR |  |  |  |
| - RVEF | rho  p | 0.28  0.35 | 0.33  0.27 |

CMR: cardiac magnetic resonance imaging; FAC: fractional area change; GLS: global longitudinal strain; RV: (systemic) right ventricle; RVEF: (systemic) right ventricular ejection fraction; TR: tricuspid regurgitation
