# Supplementary material for: Validation and Feasibility of Echocardiographic Assessment of Systemic Right Ventricular Function: Serial Correlation With MRI
Source: Front Cardiovasc Med. 2021 Mar 16;8:644193. doi: 10.3389/fcvm.2021.644193 (PMC8008818; doi:10.3389/fcvm.2021.644193)
Supplement: Supplementary file 2 [file Table_2.DOCX]

| Patient 1 | T=1 | T=2 |
| --- | --- | --- |
| CMR-RVEF (%) | 36.4 | 30.7 |
| FAC (%) | 18.3 | 20.5 |
| GLS (%) | -12.3 | -12.2 |
| Global function  (1-normal to 4-severely reduced) | 3 | 3 |
| Patient 2 |  |  |
| CMR-RVEF (%) | 46.0 | 40.1 |
| FAC (%) | 25.3 | - |
| GLS (%) | -14 | - |
| Global function  (1-normal to 4-severely reduced) | 2 | 2 |
